# Histopathological dimensions differ between aganglionic and ganglionic bowel wall in children with Hirschsprung’s disease

**DOI:** 10.1186/s12887-022-03792-3

**Published:** 2022-12-20

**Authors:** Christina Graneli, Sofia Patarroyo, Rodrigo Munoz Mitev, David Gisselsson, Emilia Gottberg, Tobias Erlöv, Tomas Jansson, Kristine Hagelsteen, Magnus Cinthio, Pernilla Stenström

**Affiliations:** 1 Department of Pediatric Surgery, Children’s Hospital, Skåne University Hospital Lund, Lund University, Lund, Sweden; 2 Department of Clinical Genetics and Pathology, Skåne University Hospital, Lund University, Lund, Sweden; 3 Department of Biomedical Engineering, Faculty of Engineering, LTH, Lund University, Lund, Sweden; 4 Department of Biomedical Engineering, Department of Clinical Engineering, Clinical Sciences Lund, LTH, Lund University, Skåne University Hospital, Lund, Sweden

**Keywords:** Hirchsprung’s disease, Bowel wall, Histopathology, Aganglionosis, Ganglionosis, Children

## Abstract

**Background:**

In the validation of new imaging technology for children with Hirschsprung’s disease (HSCR), basic anatomical parameters of the bowel wall must be established specifically for this patient group.

**Aim:**

To explore differences in histoanatomical layers of bowel wall, comparing ganglionic and aganglionic bowel walls, and to examine if the bowel wall thickness is linked to patient weight.

**Methods:**

This was an observational study of bowel specimens from children weighing 0–10 kg, operated on consecutively during 2018–2020. Ganglionic and aganglionic bowel walls were measured in digitalized microscopy images from 10 sites per trans-sectional specimen and compared regarding the thickness of their histoanatomical layers.

**Results:**

Bowel walls were measured in 21 children. Full bowel wall thickness did not differ between aganglionic and ganglionic bowel (2.20 vs 2.04; *p* = 0.802) while weight at surgery correlated positively with both ganglionic and aganglionic bowel wall thickness (*r* = 0.688 and 0.849, respectively), and age at surgery with ganglionic bowel wall thickness (*r* = 0.517). In aganglionic segments, the muscularis externa layer was thicker compared to that in ganglionosis (0.45 vs 0.31 mm, *p* = 0.012) whereas the muscularis interna was thinner (0.45 vs 0.62 mm, *p* < 0.001). A diagnostic index was identified whereby a lower ratio of muscularis interna/externa thickness followed by a thinner muscularis interna differed between aganglionic and ganglionic bowel in all specimens.

**Conclusion:**

Thicknesses of the bowel wall’s muscle layers differ between aganglionic and ganglionic bowel walls in children with HSCR. These findings support a diagnostic index that could be validated for transfer to instant diagnostic imaging techniques.

**Level of evidence:**

Diagnostic: 3

**Supplementary Information:**

The online version contains supplementary material available at 10.1186/s12887-022-03792-3.

## Highlights

• To confirm ganglionic bowel wall in Hirschsprung’s disease, intra-operative frozen biopsy samples are required. This means a prolonged anesthesia time and a more immediate diagnostic method is warranted.

• Muscle layers’ thicknesses differ between aganglionic and ganglionic bowel walls in children with Hirshsprung’s disease. These findings could be transferred to instant imaging techniques.

• See the attached Supplementary Figure 2

**Supplementary Information:**

The online version contains supplementary material available at 10.1186/s12887-022-03792-3.

## Introduction

Hirschsprung’s disease (HSCR), with a reported incidence of 1:5000, should be treated by surgical resection of the aganglionic segment and establishment of bowel continuity by a proper anorectal reconstruction [1, 2]. For diagnosing HSCR histo- and immunopathologically stained rectal biopsy is mandatory, and for determining resection length, i.e. confirming the presence of ganglionic bowel wall, it is necessary to analyze intra-operative frozen biopsy samples of bowel wall [3–5]. The intra-operative waiting time for the results of frozen biopsy means a prolonged anesthesia time for the child, which is a particular problem if repeated biopsies are required. A faster, more precise and immediate diagnostic method at disease level is warranted. Ultra-high frequency ultrasonography (UHFUS) using 50–70 MHz frequency enabling a resolution down to 30 μm, however at the cost of restricting imaging depth to only 5–10 mm, has shown promising results in precise diagnostics for small tissue structures [6, 7] as well as in a preliminary report on bowel wall in HSCR, where the histopathological layers (muscularis interna, externa and myenteric layer) have been reported to appear differently in aganglionic compared to ganglionic bowel wall [8]. For developing and validating UHFUS as a diagnostic method in HSCR, knowledge of disease-specific histopathological effects possibly caused by an absence of ganglia cells is needed. The long-term goal is to replace intraoperative biopsies or even primary diagnostic biopsy with UHFUS diagnostics. Therefore, it is worth exploring whether any differences in thickness of bowel wall layers in aganglionic versus ganglionic bowel wall can be found. Such findings could be useful in the creation of an algorithm for UHFUS diagnostics in HSCR.

### Aim and research questions

The main aim of this study was to explore whether histopathological layers of bowel wall differ regarding thickness, comparing ganglionic and aganglionic formalin-fixed and immunohistochemically stained bowel specimens from children operated on for recto-sigmoidal HSCR. The secondary aim was to explore whether the full bowel wall thicknesses of aganglionic and ganglionic bowel wall are linked with the weight of children with HSCR. This is since the UHFUS access of various histopathological layers depend on the depth and thickness of full bowel wall.

## Material and methods

### Settings

This was an observational morphometric study performed on formalin-fixed paraffin-embedded and hematoxylin–eosin stained specimens of resected bowel walls from children with HSCR. The study was conducted at one of Sweden’s two pediatric surgery national referral centers for HSCR covering an area of 5 million residents. Constituting a part of a larger project about precision diagnostics in HSCR, exploring ultra-high-resolution imaging with UHFUS, the study was carried out in collaboration with pediatric pathologists and biomedical engineers.

### Tissue samples

Bowel specimens from all children diagnosed and operated on for recto-sigmoidal HSCR between June 2018 to July 2020 were included. Inclusion criteria were children weighing less than 10 kg at the time of surgery, without prior stoma, and those who had recto-sigmoid aganglionosis stretching for a maximum of 30 cm, measured using a formalin-preserved specimen. This was to keep the group as homogenous as possible. Also, since greater body weights are often associated with delayed diagnosis, this could potentially influence the histopathological findings as a result of longer obstruction periods. Information about age and weight at surgery and bowel resection lengths was retrieved prospectively from the local HSCR register.

Prior to surgery, according to our local work-up regimen at the time of diagnosis, all patients had contrast enemas. The surgeries were performed as transendorectal pull through with a rectal cuff of 1.5–2 cm. All children had, according to the department’s routine practise, at least three, or more frequently if needed, daily regular wash outs while waiting for surgery.

In the operating theater the fresh bowel specimen was pinned on a cork mat and preserved in formalin. At the Department of Pathology the bowel was carefully cut and sectioned at every centimeter along its length. Then formalin-fixed paraffin-embedded specimens of resected bowel were analyzed for thickness of the whole bowel wall and its histological sublayers, at cross-sectional sites of ganglionic and aganglionic bowel, respectively. For study purposes cross-sectional (circular bowel) bowel images were assessed. Every cross-section specimen was judged visually as to whether it had been cross cut correctly and if it was of high quality. If skewed, the specimen was re-cut and replaced.

According to the standardized histo- and immunohistochemical staining program, hematoxylin–eosin staining was used as the primary tissue stain, to highlight cell- and tissue structures, and immunohistochemical marking with S100 and calretinin were then added to evaluate thickness and density of nerve fibers and to identify ganglion cells in the myenteric and submucosal layers [9–12]. Ganglionic bowel measurements were compared dichotomously to those of aganglionic bowel. Each patient served as her/his own control in statistical comparisons regarding histoanatomical layers.

### Morphometrical methods

Morphometrical thickness analysis of the samples was carried out using the data system Laboratory Information Management System (LIMS) RS Pathology. The tissue layers measured in both ganglionic and aganglionic bowel samples were: full bowel wall, serosa/adventitia, muscularis propria externa (the longitudinal muscle, from here called muscularis externa), muscularis propria interna (the circular muscle, from here called muscularis interna), myenteric tissue layer (located in between the muscularis propria layers including nerve trunks and extracellular matrix), and the submucosa and mucosa.

**Fig. 1:**
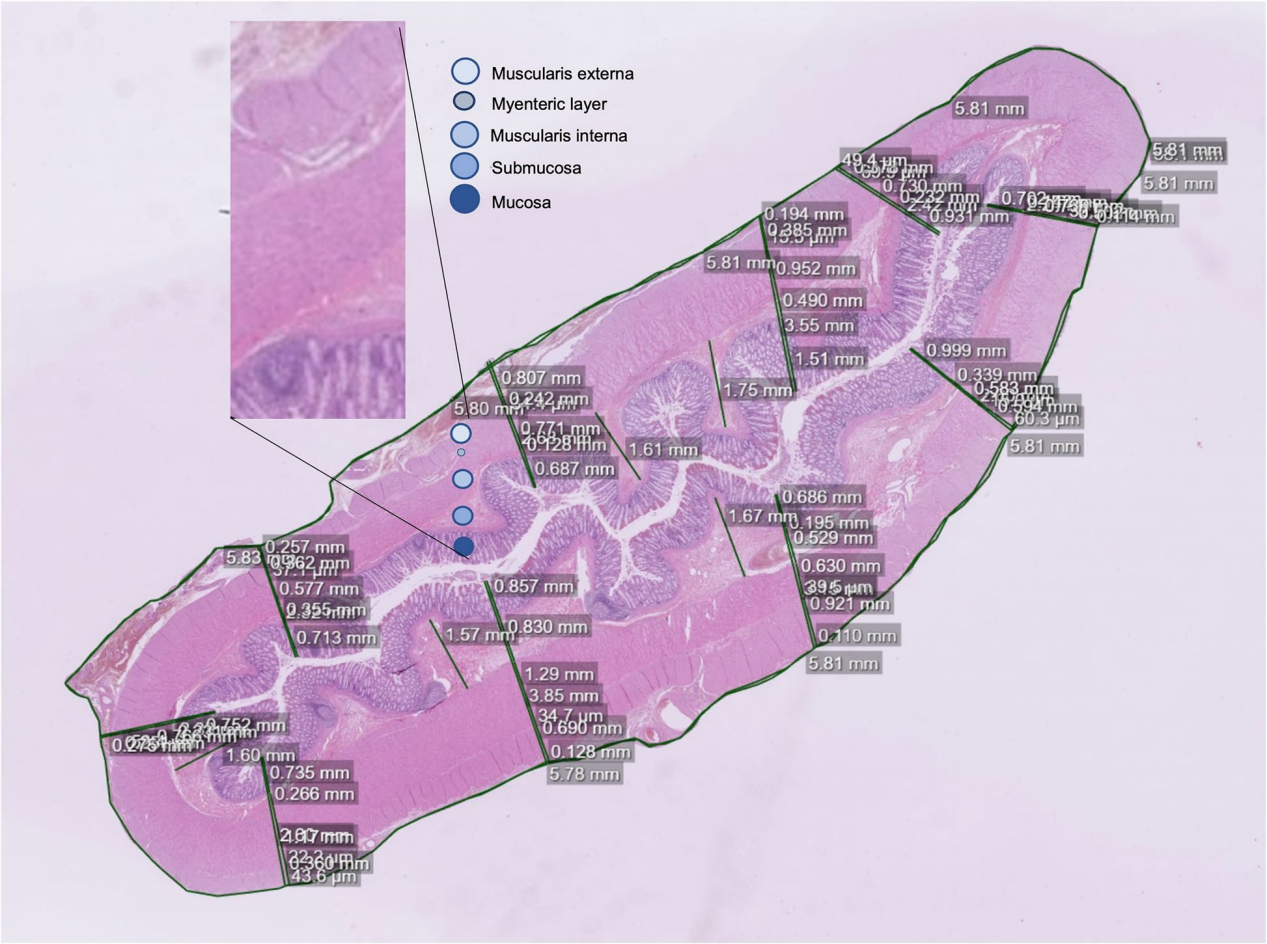
Ten scattered measurements and five measurements of the highest submucosal amplitudes on a tissue sample. The median thickness of each histopathological layer and the median thickness of five of the submucosa’s thickest sites were calculated

To obtain as objective measurements as possible, each bowel section was measured at 10 points, and means of the 10 measurements of full bowel wall and each histopathological layer were used in statistical comparisons (Fig. 1). The interspace between the 10 points was selected by dividing the circumference of each sample by 10. In order to investigate the interrelationship between the thicknesses of the muscularis interna and externa in each patient, the ratio of muscularis interna/muscularis externa was calculated. Since the thickness of the submucosa appeared to vary greatly, it was also measured at the five thickest points, measured between the mucosa’s and muscularis interna’s inner circumferences (Fig. 1). Thicknesses were described in millimeters (mm). Measurements were made by one of the authors (SP) and a quality control was undertaken by a pathologist (RM) using computer-saved assessments.

### Statistical analysis

Statistical analysis was performed using Microsoft® Excel and IBM® SPSS® Statistics version 26. The statistical design was assisted by statisticians at Clinical Studies Forum South.

Descriptive statistics and boxplots were displayed in median, range and interquartile ranges.

Distribution of measurements was tested in scatter diagrams and histograms and non-parametric methods selected due to unequal distribution. Median values for the paired Wilcoxon signed test were used in the dichotomous comparisons. For correlation between data of bowel wall thickness, weight and age, the Spearman correlation was used. A p-value < 0.05 was considered to be statistically significant.

### Ethical consideration

Approval for the study was obtained from the regional ethics review board (Dnr: 2017/769) Parental consent for participation was obtained.

## Results

### Patients and specimen characteristics

During the study period, 28 children were operated on for HSCR. Six were excluded from the study because of aganglionosis stretching longer than 30 cm and one as a result of the weight being over 10 kg, leaving 21 resected rectosigmoid bowel specimens for analysis. The median resected length after fixation in formalin was 19 cm (range 10–30 cm) and the transition zone was a median of 3 cm (2–6 cm). The gestational weight of the children was a median of 3.3 kilos (2.1–4.2), and their gestational age was 39 weeks (35–41 weeks); three were born prematurely before week 38. No child in the cohort had Trisomy 21. Their age and weight at the time of the surgery was a median of 23 days (10–480) and 3.8 kg (2.6–9.8), respectively. The majority of children were boys (20/21; 95%). All included children had primary pull through surgery without previous stomas, and all had at least three daily regular wash outs, or more when needed, while waiting for surgery. No child’s colon presented with severe dilatation during surgery and no child ended up with a protective stoma due to bowel dilatation.

### Bowel wall full thickness

Serving as their own controls, bowel wall thickness of the operated children did not differ significantly between aganglionic and ganglionic bowel in paired statistical tests (Table 1, Supplementary).

**Table 1:** Histo-anatomical thicknesses comparing aganglionic versus ganglionic bowel wall in 21 children with Hirschsprung’s disease

Histo-anatomical layers	Thickness aganglionosis (millimeters)	Thickness ganglionosis (millimeters)	*p*-value*	Difference between aganglionic versus ganglionic bowel (%)	Thinner in aganglionosis versus ganglionosis (n)
Full bowel wall	2.20 (1.26–3.98)	2.04 (1.66–3.10)	0.802	5 (-42–59)	10
Serosa and subserosa	0.14 (0.02–0.48)	0.19 (0.04–0.46)	0.849	-16 (-79–325)	12
Muscularis externa	0.45 (0.15–1.60)	0.31 (0.21–0.49)	***0.012***	75 (-48–261)	9
Muscularis interna	0.45 (0.29–1.02)	0.62 (0.43–0.97)	** < ** ***0.001***	-33 (-54–4)	19
Ratio muscularis interna/externa	1.00 (0.42–2.34)	2.07 (1.60–3.40)	** < ** ***0.001***	-50 (-88–4)	20 (lower)
Myenteric layer	0.03 (0.006–0.15)	0.03 (0.007–0.04)	0.050	50 (-77–430)	8
Submucosa	0.30 (0.17 -1.27)	0.34 (0.18–0.85)	0.500	0 (-66–182)	10
Submucosa maximum thickness^a^	1.33 (0.46–2.11)	1.10 (0.76–1.40)	0.571	21 (-53–149)	7
Mucosa	0.52 (0.31–1.03)	0.52 (0.37–0.84)	0.667	-5 (-54–100)	11

Median (range)

^*^Wilcoxon paired signed

^a^median thickness of the thickest five measurements

**Fig. 2:**
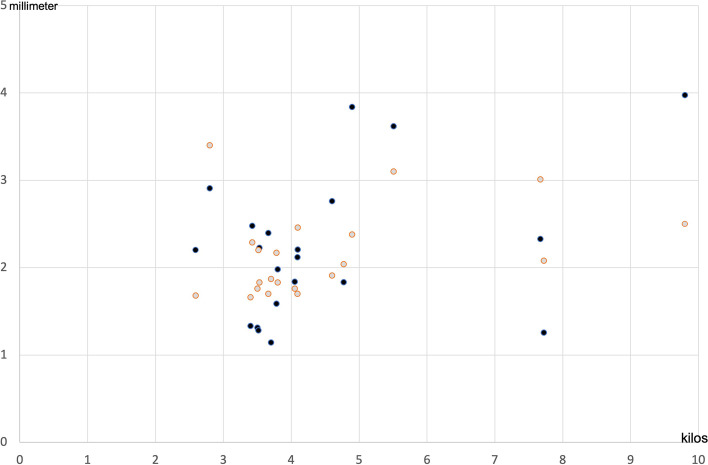
Correlation between aganglionic respective ganglionic full thickness bowel wall and children’s weights at surgery for Hirschsprung’s disease. Ganglionic bowel wall thickness (black spots) *r* = 0.669*; *p* < 0.001 Aganglionc bowel wall thickness (gray spots) *r* = 0.849*; *p* < 0.001 *Spearman correlation test

On group level analyzes, both in aganglionic and ganglionic bowel, the thickness of full bowel wall correlated significantly with the children’s weight at the time of surgery (Spearman correlation, *p* < 0.001 respectively; Fig. 2) and also with age for ganglionic bowel (Spearman correlation *r* = 0.517; *p* = 0.016). There was no significant correlation between the extension of disease (length of the aganglionosis) and the bowel wall thickness (Spearman correlation *r* = 0.003, *p* = 0.989).

### Bowel walls’ histological layers

**Fig. 3:**
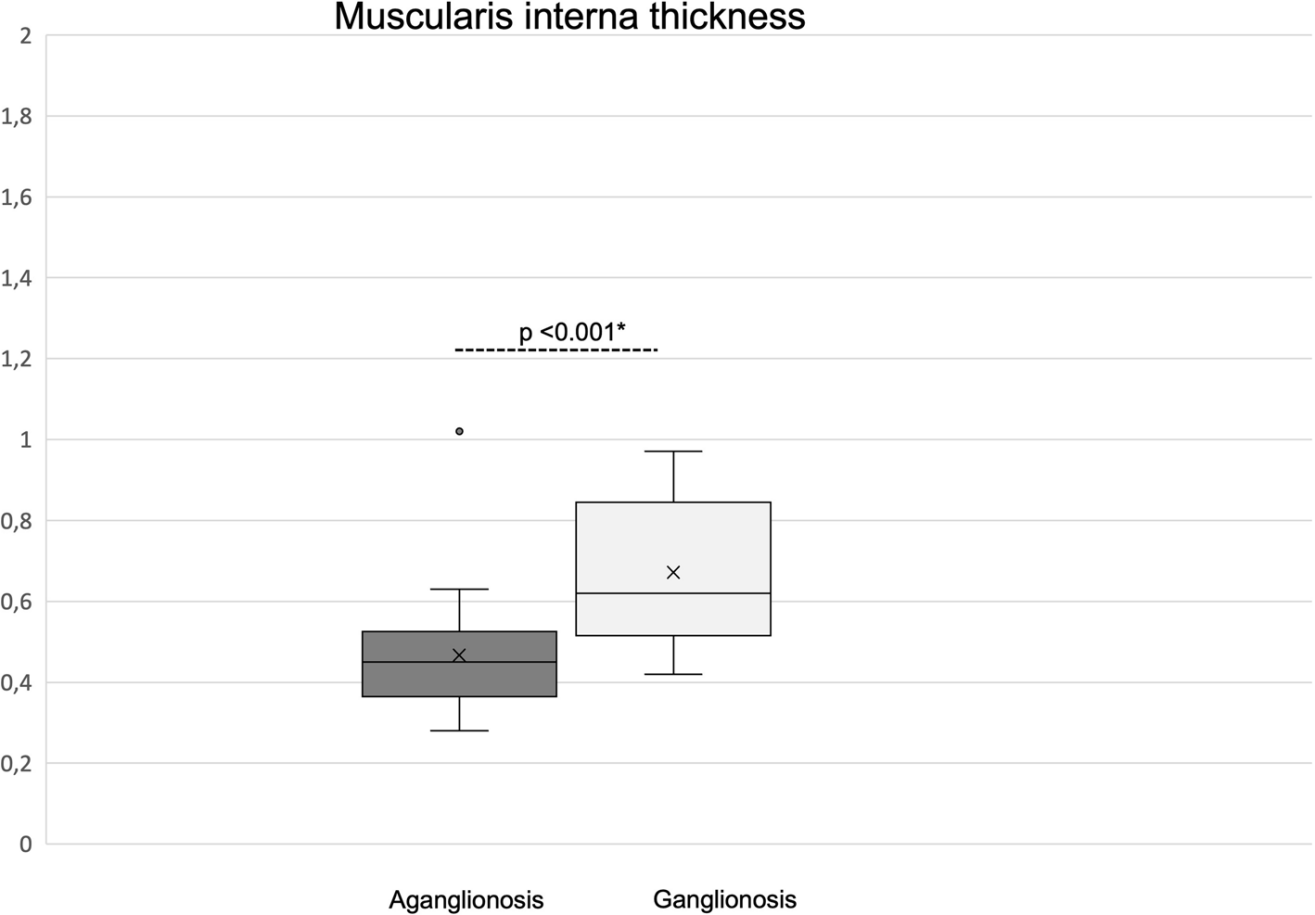
Thickness of muscularis externa in aganglionosis versus ganglionosis. *Paired Wilcoxon

**Fig. 4:**
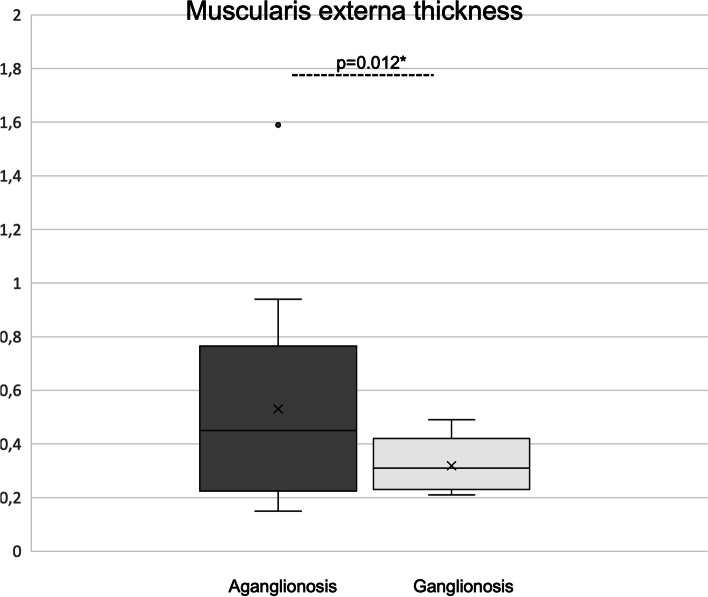
Thickness of muscularis interna in aganglionosis versus ganglionosis. *Paired Wilcoxon

In aganglionic bowel, compared to ganglionic bowel, the muscularis interna was significantly thinner (0.45 mm versus 0.62 mm; *p* < 0.001) and the muscularis externa significantly thicker (0.45 mm versus 0.31 mm; *p* = 0.012) (Table 1). The muscularis interna was thinner in aganglionosis in 19/21 while in the two other children (2/21), the muscularis interna was limited to be 4% thicker in aganglionic bowel compared to that in ganglionic bowel. While the thickness of the muscularis interna was homogenic (Fig. 3), the thickness of the muscularis externa differed widely between patients (Fig. 4). The thickness difference between aganglionosis and ganglionosis in the muscularis externa was a median of 0.17 mm (-0.16–1.42) which did not correlate to age at surgery (*r* = -0.011; *p* = 0.961).

**Fig. 5:**
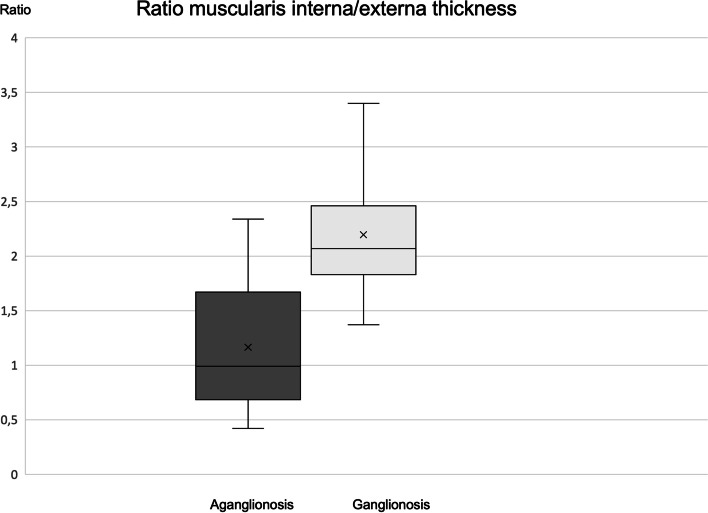
Ratio of muscularis interna/muscularis externa in aganglionosis versus ganglionosis *Paired Wilcoxon

The ratio of muscularis interna/externa thickness was significantly (-50%) lower in aganglionosis (1.00 versus 2.07; *p* < 0.001) being lower in 20/21 specimens (Table 1, Fig. 5). In the single specimen where the ratio was lower in ganglionic bowel, the difference was limited to 4%. The myenteric layer between the muscularis externa and interna was overall descriptively thicker in aganglionic bowel (50%) but significance was not reached (*p* = 0.05) and the thickness differed considerably between patients, especially in aganglionic bowel, (Table 1, Supplementary).

The median thickness of the submucosa did not differ significantly between aganglionosis and ganglionosis, and although the submucosa’s maximum thickness (median of the five thickest sites) was overall 21% higher in aganglionic compared to ganglionic bowel, the thickness varied greatly and did not reach any statistical difference, and (Table 1, Supplementay).

### Diagnostic algorithm and dimensional distribution

Based on these findings, a diagnostic index for aganglionosis was identified: 1. A lower thickness ratio of muscularis interna/externa followed by 2. A thinner muscularis interna, indicated the presence of aganglionosis in all specimens. The index was anchored in following: the one specimen with a higher ratio muscularis interna/externa in aganglionosis still showed a thinner muscularis interna in aganglionosis. The reverse was also true: the two specimens with thicker muscularis interna in aganglionosis than in ganglionosis both showed a lower ratio of interna/externa thickness in aganglionosis.

**Fig. 6:**
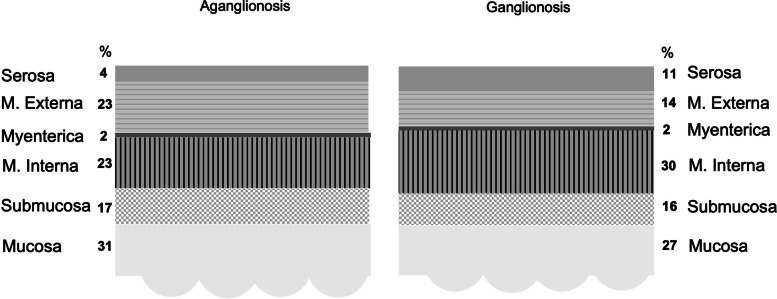
Distribution (%) of thickness of bowel walls’ histopathological layers in aganglionic and ganglionic bowel wall, respectively. Aganglionosis *n* = 21, with a median bowel wall thickness of 2.15 mm. Ganglionosis *n* = 21, with a median bowel wall thickness of 2.03 mm

A dimensional distribution (%) of the different histopathological layers in aganglionic and ganglionic bowel walls is displayed in Fig. 6.

## Discussion

The results of the study show that, in histopathological prepared bowel specimens, the thickness of the muscularis interna was thinner, the muscularis externa thicker and the ratio of the interna/externa thickness was greater in aganglionic bowel wall. In addition, the myenteric layer, where the myenteric nerve plexus is found in normal bowel wall, tended to be thicker in aganglionic bowel wall but the thickness varied greatly especially in aganglionosis. The thickness of the whole bowel wall did not differ between aganglionic and ganglionic bowel wall, and both ganglionic and aganglionic bowel wall thickness increased with the patient’s weight, i.e. the thickness of the bowel wall seemed to grow with the patient regardless of the presence or absence of ganglion cells.

The cardinal histological feature of bowel wall in HSCR patients is the absence of ganglion cells in the myenteric layer and in the submucosa [10, 11, 13]. However, secondary effects of aganglionosis, such as histopathological dimensional differences, become relevant when exploring new diagnostic opportunities such as UHFUS in HSCR diagnostics [8]. To study the muscle thicknesses of a developing organ, e.g. the gut, in the presence of a complex, highly variable condition such as HSCR, is challenging and dimensional differences in the bowel walls between aganglionic versus ganglionic bowel have, to date, to the authors’ best knowledge, only been described in murine models [14, 15]. In one of those, when only long segment HSCR was studied, both the muscle layers were thicker in aganglionic bowel [14]. In the other study both layers were thicker in ganglionic bowel [15]. The findings were explained speculatively as hypertonicity with an increased number of muscle cells in the muscularis interna in aganglionosis [16] resulting in the development of a muscle hypertrophy due to a high working force against a contracted aganglionic segment [15]. These speculations were contradictory to our results in which we showed that the muscularis interna was thinner and the externa thicker when ganglion cells were absent. Reasons for these differences can only be speculated upon. The most obvious differences between the former studies and ours are that the former studies were performed on animal models, they included total colonic aganglionosis and that they explored bowel exposed for bowel obstruction without decompressing treatment (wash outs). In addition none of them reported on the two bowel wall’s muscle layers separately. This was in contrast to our study which exclusively explored human bowel wall with only rectosigmoid aganglionosis in children who had their obstruction decompressed before surgical resection, and that our study explored the muscularis interna and externa as separate anatomical structures. Speculatively the regular wash outs for decompression might have impacted on the thickness of the bowel wall, and maybe also the interrelationship between the muscularis interna and externa.

In our study the myenteric layer tended to be more prominent in aganglionic bowel, but the results differed considerably between patients. Also, in our UHFUS preliminary report a more prominent and uneven myenteric layer was described in imaged aganglionosis [8]. An increased thickness of this layer might speculatively and, according to some literature, be caused by hypertrophy of nerve trunks and/or increased deposition of extracellular matrix replacing the absent ganglia cells in aganglionosis [17–19]. Such transition could vary over time and individually, or the layer’s appearance might depend on the thickness of the surrounding tissue (muscularis interna and externa), which might explain its variation. More detailed studies of the appearance of the myenteric layer on UHFUS are currently being undertaken by our research group.

In our pilot assessments it seemed that the height of submucosal amplitudes differed between aganglionotic and ganglionotic bowel. However, after multiple measurements in our main calculations and analyses, we could not verify such a difference statistically. Instead the submucosa varied greatly within each patient, and especially in aganglionic specimens. This could, speculatively, depend on the fact that aganglionosis to a larger extent can be present with various amounts of fibrosis or collagen deposits in different parts, e.g. when replacing ganglia cells. This issue is not solved and constitutes a research question in our current project.

Extrapolating the histopathological findings, presented here, to the developmental process of UHFUS in HSCR diagnostics, the capabilities of UHFUS need to be considered. The transducer of 50–70 MHz image has an optimal depth of only 10 mm and facilitates a resolution down to 30 µm [6, 7, 20]. This could be compared to the most frequently used ultrasound transducers with delivery frequencies of 2–18 MHz, capturing image depths of several cm [20] which is not suitable for a detailed exposure of the bowel wall. According to the histopathological morphometric results, the muscularis interna and externa thicknesses ranged from 0.15–1.60 mm on a depth of 2–3 mm from the serosa. Therefore, and in line with our UHFUS report [8], the UHFUS is quite capable of enabling imaging of the muscularis interna and externa. However, a more uncertain histopathological layer for UHFUS assessment might be the myenteric layer. According to our results, the myenteric layer ranged from 6 to 150 µm which means that some specimens had a myenteric layer thickness too thin for UHFUS assessment, although the myenteric layer is located at a reasonable depth of about 0.5 mm from the serosa. The submucosa was sometimes found at a depth of 3–5 mm, so if the signal becomes too weak at this depth, the 30–50 MHz UHFUS-transducer to probe deeper can be used instead, or the submucosa can be assessed from a mucosal approach. One concern that has been raised is if UHFUS in vivo examinations could be affected by colonic movements. However, in our clinical pilot studies with UHFUS during surgical procedures, the colon did not show any signs of movement at all.

Bowel wall thickness did not differ between aganglionic and ganglionic segments, and was shown to increase with the child’s weight, regardless of whether aganglionosis or ganglionosis was present. This finding impacts the development and validation of UHFUS in HSCR diagnostics, in terms of whether the same transducer frequency can be used for assessing both aganglionosis and ganglionosis. On the other hand in our study we only examined children weighing less than 10 kg, and greater weights might limit the tissue depth. Bowel wall thickness has been shown to be age dependent in healthy children examined with normo-frequent ultrasound [21, 22], but specific information on weight and bowel wall thickness are lacking, and especially in children with HSCR. In children with HSCR, it should be considered that ganglionic bowel wall could be secondarily affected by the disease, which is why our results on ganglionic bowel might not be able to be fully extrapolated to healthy children. This is supported by our finding that bowel wall thickness correlated to the time of the surgery (disease duration).

Another finding worthy of discussion, is that the thickness of the muscularis externa, in the majority of cases, was greater in ganglionosis. The absence of innervation is generally considered to diminish muscle mass, and to cause atrophy. In our results this was true for the muscularis interna, but not for the muscularis externa, at least not in most patients. Speculating, it could be that the interna and externa muscularis work as a unit: when one becomes thicker (stronger) the other gets thinner, i.e. compensating for each other. If so, the interrelationship between the interna and externa muscularis here described as the ratio, becomes important.

One strength of the study was the high quality of the tissue samples. They all contained well-preserved tissue structures of all histological layers, and together with stringent tissue handling and staining, precise differentiation of the various layers and accurate measurements were possible. A statistically relevant strength was that patients served as their own controls and therefore weight diversity did not matter for paired testing, and the paired testing increased the power of the otherwise low number of patients. Another strength was the digital imaging analysis, enabling exact consistent measurements including to three decimal places, and quality controls and re-analysis by a pathologist of the saved images. One obvious limitation was the studying of formalin-fixed specimens, when fresh bowel in vivo would be more relevant for clinical use. The histopathological preparation, including formalin treatment, has been reported to potentially alter the tissue, impacting particularly on the collagen deposits, which hypothetically could play a key role in HSCR [19] as the dimensions of the histopathological layers might be affected unequally in aganglionic versus ganglionic tissue. Such preparational effects have, to the authors’ best knowledge, never before been studied in human bowel wall but it is important to bear in mind that the histopathologic thickness may not fully correspond to that of in vivo tissue. Another limitation was the lack of control material which influences the capacity to show diagnostic efficacy compared to healthy specimens. Dimensional differences have, to our best knowledge, never been studied before in healthy human bowel but would be warranted in order to explore whether dimensional differences between the rectum and sigmoideum are evident. However, such studies on healthy children, in which the child constitutes its own control, would be difficult to undertake since healthy children very seldom require rectosigmoidal resections or sigmoid full wall biopsies. Another limitation was that the transition zone’s histopathological features with regard to histoanatomical thickness were not studied. Hypothetically the muscle layers in the transition zone could be either thinner or thicker as a result of physiological or anatomical reasons.

Despite several limitations, the histopathological results presented here will be useful and transferred to UHFUS imaging of bowel wall. In particular, the finding of the index indicating a lower thickness ratio muscularis interna/externa followed by a thinner muscularis interna will be useful. Importantly, the index here presented is only used to illustrate the interrelationship between the interna and externa muscularis in each patient. It could not be implemented clinically before several more examinations have been carried out, validating the results presented in this study. Furthermore, a very important aspect of exploring the role of UHFUS in HSCR, is that both the ultrasound examination and the expertise of the pathologist caring for the pathological material could be very in-person dependent. This means that generalization of results could be difficult, calling for a very careful interpretation.

## Conclusion

This study demonstrates that thicknesses of the bowel wall’s muscle layers differ between aganglionic and gangionic bowel wall specimens of children with rectosigmoid HSCR. The finding of a lower thickness ratio of muscularis interna/externa and a thinner muscularis interna in aganglionosis, constitutes an index to be considered when validating UHFUS in HSCR diagnostics.

## Supplementary Information

**Additional file 1.**

## Data Availability

The datasets used and analysed during the current study can be available from the corresponding author on reasonable request.

## Abbreviations

HSCR: Hirchsprung’s disease
LIMS: Laboratory Information Management System
UHFUS: Ultra-high frequency ultrasonography
