# Supplementary material for: Histopathological dimensions differ between aganglionic and ganglionic bowel wall in children with Hirschsprung’s disease
Source: BMC Pediatr. 2022 Dec 20;22:723. doi: 10.1186/s12887-022-03792-3 (PMC9764572; doi:10.1186/s12887-022-03792-3)

Histopathological dimensions differ between aganglionic and ganglionic bowel wall in children with Hirschsprung's disease

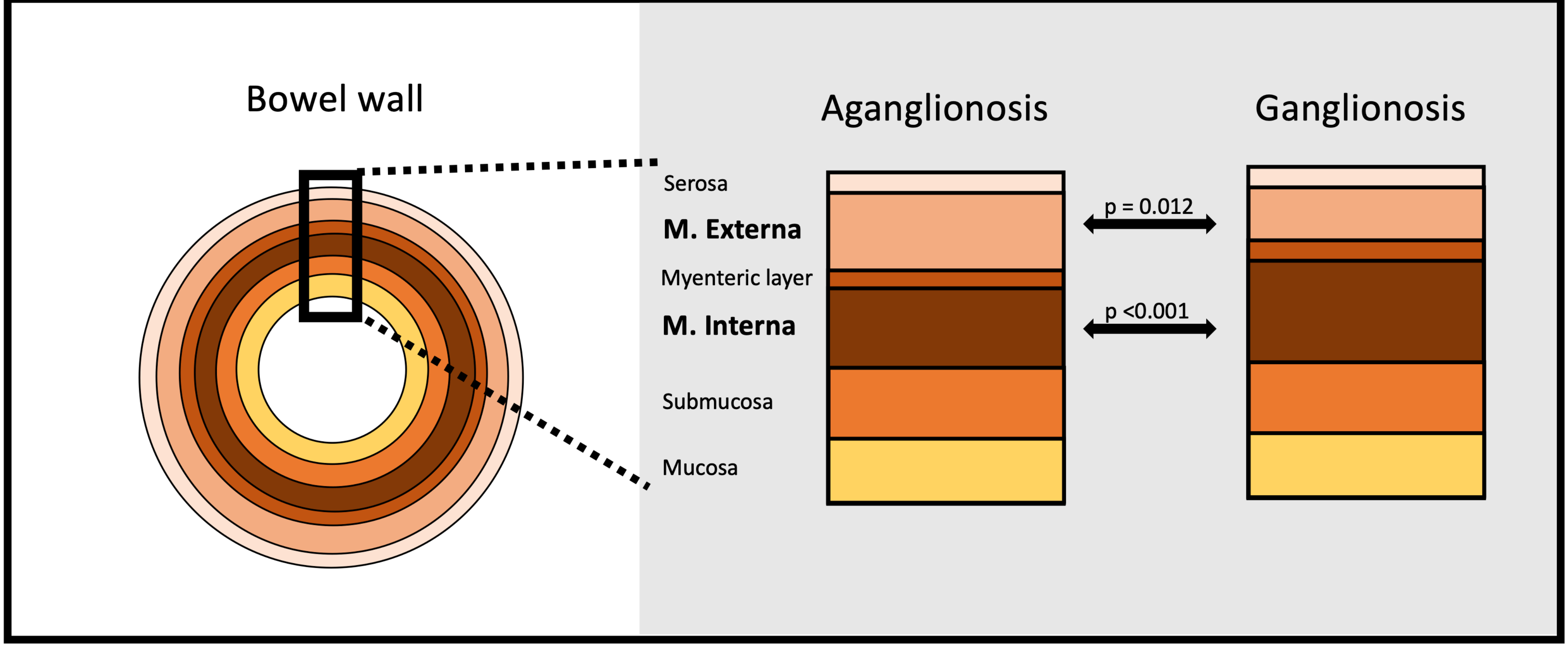

Supplement: Supplementary file 1 — Additional file 1. [file 12887_2022_3792_MOESM1_ESM.zip › 12887_2022_3792_MOESM1_ESM/Supplementary Figure 2.pdf]
